# Supplementary material for: Modeling glioblastoma heterogeneity as a dynamic network of cell states
Source: Mol Syst Biol. 2021 Sep 16;17(9):e10105. doi: 10.15252/msb.202010105 (PMC8444284; doi:10.15252/msb.202010105)
Supplement: Supplementary file 6 — Source Data for Figure 5 [file MSB-17-e10105-s004.zip › Figure5A_sourcedata/GSEA_3071/hallmarks_stateII.GseaPreranked.1623742536692/HALLMARK_G2M_CHECKPOINT.html]

Details for gene set HALLMARK\_G2M\_CHECKPOINT[GSEA]

|  || Dataset | state23071 |
| Phenotype | NoPhenotypeAvailable |
| Upregulated in class | na\_pos |
| GeneSet | HALLMARK\_G2M\_CHECKPOINT |
| Enrichment Score (ES) | 0.6310914 |
| Normalized Enrichment Score (NES) | 3.991212 |
| Nominal p-value | 0.0 |
| FDR q-value | 0.0 |
| FWER p-Value | 0.0 |
Table: GSEA Results Summary

  

Fig 1: Enrichment plot: HALLMARK\_G2M\_CHECKPOINT      
 Profile of the Running ES Score & Positions of GeneSet Members on the Rank Ordered List

  

| PROBE | GENE SYMBOL | GENE\_TITLE | RANK IN GENE LIST | RANK METRIC SCORE | RUNNING ES | CORE ENRICHMENT || 1 | CDC6 |  |  | 11 | 1.631 | 0.0141 | Yes |
| 2 | CDK1 |  |  | 14 | 1.540 | 0.0296 | Yes |
| 3 | BRCA2 |  |  | 22 | 1.445 | 0.0428 | Yes |
| 4 | UBE2C |  |  | 23 | 1.438 | 0.0577 | Yes |
| 5 | MYBL2 |  |  | 28 | 1.362 | 0.0708 | Yes |
| 6 | FBXO5 |  |  | 30 | 1.359 | 0.0846 | Yes |
| 7 | ORC6 |  |  | 39 | 1.308 | 0.0962 | Yes |
| 8 | GINS2 |  |  | 40 | 1.306 | 0.1097 | Yes |
| 9 | AURKB |  |  | 44 | 1.276 | 0.1222 | Yes |
| 10 | CCNA2 |  |  | 53 | 1.230 | 0.1329 | Yes |
| 11 | CDC45 |  |  | 59 | 1.212 | 0.1442 | Yes |
| 12 | MAD2L1 |  |  | 61 | 1.199 | 0.1564 | Yes |
| 13 | TOP2A |  |  | 72 | 1.132 | 0.1656 | Yes |
| 14 | KIF2C |  |  | 73 | 1.132 | 0.1774 | Yes |
| 15 | MCM5 |  |  | 74 | 1.128 | 0.1890 | Yes |
| 16 | RBL1 |  |  | 82 | 1.110 | 0.1988 | Yes |
| 17 | NDC80 |  |  | 94 | 1.074 | 0.2071 | Yes |
| 18 | POLA2 |  |  | 95 | 1.068 | 0.2182 | Yes |
| 19 | TMPO |  |  | 99 | 1.056 | 0.2284 | Yes |
| 20 | MCM3 |  |  | 105 | 1.035 | 0.2379 | Yes |
| 21 | MKI67 |  |  | 107 | 1.031 | 0.2483 | Yes |
| 22 | STIL |  |  | 108 | 1.031 | 0.2590 | Yes |
| 23 | TPX2 |  |  | 111 | 1.025 | 0.2691 | Yes |
| 24 | KIF11 |  |  | 115 | 1.014 | 0.2789 | Yes |
| 25 | SMC2 |  |  | 123 | 1.005 | 0.2875 | Yes |
| 26 | CHAF1A |  |  | 127 | 0.999 | 0.2971 | Yes |
| 27 | CKS1B |  |  | 128 | 0.999 | 0.3075 | Yes |
| 28 | TACC3 |  |  | 130 | 0.992 | 0.3175 | Yes |
| 29 | KIF20B |  |  | 133 | 0.989 | 0.3272 | Yes |
| 30 | CDK4 |  |  | 137 | 0.986 | 0.3367 | Yes |
| 31 | EXO1 |  |  | 145 | 0.969 | 0.3450 | Yes |
| 32 | E2F1 |  |  | 153 | 0.951 | 0.3531 | Yes |
| 33 | KIF23 |  |  | 161 | 0.936 | 0.3610 | Yes |
| 34 | RPA2 |  |  | 169 | 0.929 | 0.3689 | Yes |
| 35 | MCM6 |  |  | 176 | 0.906 | 0.3767 | Yes |
| 36 | KPNB1 |  |  | 186 | 0.891 | 0.3837 | Yes |
| 37 | PLK4 |  |  | 205 | 0.870 | 0.3882 | Yes |
| 38 | TFDP1 |  |  | 209 | 0.866 | 0.3964 | Yes |
| 39 | PRIM2 |  |  | 212 | 0.865 | 0.4049 | Yes |
| 40 | PBK |  |  | 214 | 0.864 | 0.4136 | Yes |
| 41 | TLE3 |  |  | 215 | 0.861 | 0.4225 | Yes |
| 42 | EZH2 |  |  | 233 | 0.839 | 0.4269 | Yes |
| 43 | HMGA1 |  |  | 238 | 0.834 | 0.4345 | Yes |
| 44 | DBF4 |  |  | 240 | 0.832 | 0.4429 | Yes |
| 45 | RACGAP1 |  |  | 241 | 0.831 | 0.4515 | Yes |
| 46 | PRC1 |  |  | 242 | 0.830 | 0.4601 | Yes |
| 47 | LMNB1 |  |  | 251 | 0.824 | 0.4667 | Yes |
| 48 | CHEK1 |  |  | 252 | 0.822 | 0.4752 | Yes |
| 49 | DKC1 |  |  | 268 | 0.789 | 0.4796 | Yes |
| 50 | SMC4 |  |  | 279 | 0.777 | 0.4851 | Yes |
| 51 | CENPE |  |  | 284 | 0.775 | 0.4921 | Yes |
| 52 | BARD1 |  |  | 295 | 0.760 | 0.4975 | Yes |
| 53 | CENPA |  |  | 303 | 0.751 | 0.5035 | Yes |
| 54 | POLE |  |  | 305 | 0.750 | 0.5110 | Yes |
| 55 | NSD2 |  |  | 308 | 0.747 | 0.5182 | Yes |
| 56 | KIF4A |  |  | 309 | 0.746 | 0.5260 | Yes |
| 57 | CKS2 |  |  | 312 | 0.745 | 0.5332 | Yes |
| 58 | CCNF |  |  | 321 | 0.737 | 0.5388 | Yes |
| 59 | AURKA |  |  | 360 | 0.705 | 0.5365 | Yes |
| 60 | DTYMK |  |  | 364 | 0.701 | 0.5431 | Yes |
| 61 | TTK |  |  | 365 | 0.701 | 0.5503 | Yes |
| 62 | UCK2 |  |  | 374 | 0.695 | 0.5555 | Yes |
| 63 | MT2A |  |  | 393 | 0.682 | 0.5580 | Yes |
| 64 | KIF15 |  |  | 404 | 0.676 | 0.5625 | Yes |
| 65 | KPNA2 |  |  | 420 | 0.669 | 0.5657 | Yes |
| 66 | ODC1 |  |  | 430 | 0.664 | 0.5703 | Yes |
| 67 | INCENP |  |  | 432 | 0.660 | 0.5769 | Yes |
| 68 | BUB3 |  |  | 441 | 0.657 | 0.5816 | Yes |
| 69 | SLC7A1 |  |  | 448 | 0.651 | 0.5869 | Yes |
| 70 | RAD54L |  |  | 456 | 0.648 | 0.5918 | Yes |
| 71 | HMMR |  |  | 475 | 0.639 | 0.5939 | Yes |
| 72 | KNL1 |  |  | 504 | 0.628 | 0.5934 | Yes |
| 73 | BIRC5 |  |  | 505 | 0.628 | 0.5999 | Yes |
| 74 | SNRPD1 |  |  | 523 | 0.615 | 0.6020 | Yes |
| 75 | DDX39A |  |  | 542 | 0.605 | 0.6037 | Yes |
| 76 | NOLC1 |  |  | 546 | 0.603 | 0.6092 | Yes |
| 77 | HSPA8 |  |  | 547 | 0.603 | 0.6155 | Yes |
| 78 | CDC25A |  |  | 572 | 0.589 | 0.6155 | Yes |
| 79 | CENPF |  |  | 575 | 0.588 | 0.6211 | Yes |
| 80 | MAP3K20 |  |  | 587 | 0.583 | 0.6244 | Yes |
| 81 | SMC1A |  |  | 618 | 0.570 | 0.6227 | Yes |
| 82 | NCL |  |  | 638 | 0.560 | 0.6237 | Yes |
| 83 | NUSAP1 |  |  | 654 | 0.550 | 0.6256 | Yes |
| 84 | SLC38A1 |  |  | 656 | 0.550 | 0.6311 | Yes |
| 85 | MCM2 |  |  | 734 | 0.521 | 0.6171 | No |
| 86 | NASP |  |  | 741 | 0.518 | 0.6209 | No |
| 87 | POLQ |  |  | 771 | 0.508 | 0.6189 | No |
| 88 | MYC |  |  | 790 | 0.502 | 0.6195 | No |
| 89 | SRSF10 |  |  | 831 | 0.489 | 0.6145 | No |
| 90 | CDC20 |  |  | 844 | 0.486 | 0.6165 | No |
| 91 | NUP50 |  |  | 888 | 0.473 | 0.6106 | No |
| 92 | SUV39H1 |  |  | 925 | 0.464 | 0.6063 | No |
| 93 | SRSF2 |  |  | 941 | 0.461 | 0.6073 | No |
| 94 | CDC7 |  |  | 971 | 0.451 | 0.6047 | No |
| 95 | KIF22 |  |  | 975 | 0.450 | 0.6086 | No |
| 96 | PLK1 |  |  | 1009 | 0.442 | 0.6049 | No |
| 97 | CASP8AP2 |  |  | 1025 | 0.436 | 0.6056 | No |
| 98 | NEK2 |  |  | 1100 | 0.417 | 0.5913 | No |
| 99 | BUB1 |  |  | 1145 | 0.408 | 0.5844 | No |
| 100 | AMD1 |  |  | 1166 | 0.403 | 0.5836 | No |
| 101 | HNRNPD |  |  | 1177 | 0.401 | 0.5852 | No |
| 102 | FANCC |  |  | 1186 | 0.399 | 0.5873 | No |
| 103 | STMN1 |  |  | 1249 | 0.387 | 0.5757 | No |
| 104 | ESPL1 |  |  | 1255 | 0.386 | 0.5784 | No |
| 105 | NUP98 |  |  | 1314 | 0.375 | 0.5677 | No |
| 106 | DR1 |  |  | 1352 | 0.366 | 0.5622 | No |
| 107 | TRAIP |  |  | 1430 | 0.351 | 0.5464 | No |
| 108 | SLC7A5 |  |  | 1434 | 0.351 | 0.5493 | No |
| 109 | ORC5 |  |  | 1556 | 0.331 | 0.5222 | No |
| 110 | CDC27 |  |  | 1562 | 0.330 | 0.5243 | No |
| 111 | TENT4A |  |  | 1593 | 0.325 | 0.5201 | No |
| 112 | EFNA5 |  |  | 1624 | 0.320 | 0.5159 | No |
| 113 | G3BP1 |  |  | 1661 | 0.315 | 0.5101 | No |
| 114 | HIF1A |  |  | 1667 | 0.315 | 0.5121 | No |
| 115 | MTF2 |  |  | 1710 | 0.309 | 0.5047 | No |
| 116 | SLC12A2 |  |  | 1712 | 0.309 | 0.5077 | No |
| 117 | CCND1 |  |  | 1753 | 0.304 | 0.5007 | No |
| 118 | RAD23B |  |  | 1777 | 0.301 | 0.4980 | No |
| 119 | KMT5A |  |  | 1787 | 0.300 | 0.4989 | No |
| 120 | ATF5 |  |  | 1794 | 0.299 | 0.5005 | No |
| 121 | ODF2 |  |  | 1887 | 0.287 | 0.4803 | No |
| 122 | E2F3 |  |  | 1961 | 0.277 | 0.4647 | No |
| 123 | CUL4A |  |  | 2032 | 0.269 | 0.4499 | No |
| 124 | RBM14 |  |  | 2103 | 0.262 | 0.4349 | No |
| 125 | CBX1 |  |  | 2210 | 0.251 | 0.4108 | No |
| 126 | SMARCC1 |  |  | 2837 | -0.342 | 0.2565 | No |
| 127 | MARCKS |  |  | 3369 | -0.487 | 0.1277 | No |
| 128 | ATRX |  |  | 3509 | -0.546 | 0.0983 | No |
| 129 | ARID4A |  |  | 3558 | -0.576 | 0.0921 | No |
| 130 | MEIS1 |  |  | 3651 | -0.648 | 0.0757 | No |
| 131 | FOXN3 |  |  | 3671 | -0.661 | 0.0777 | No |
| 132 | CDKN1B |  |  | 3812 | -0.800 | 0.0507 | No |
| 133 | MEIS2 |  |  | 4073 | -2.041 | 0.0063 | No |
Table: GSEA details [plain text format]

  

Fig 2: HALLMARK\_G2M\_CHECKPOINT: Random ES distribution      
 Gene set null distribution of ES for **HALLMARK\_G2M\_CHECKPOINT**
